# Ecological Impacts of Exotic Species on Native Seed Dispersal Systems: A Systematic Review

**DOI:** 10.3390/plants12020261

**Published:** 2023-01-05

**Authors:** Sebastián Cordero, Francisca Gálvez, Francisco E. Fontúrbel

**Affiliations:** 1 Instituto de Biología, Facultad de Ciencias, Pontificia Universidad Católica de Valparaíso, Valparaíso 2373223, Chile; 2 Departamento de Botánica, Universidad de Concepción, Concepción 4030000, Chile; 3 Rizoma, Centro de Estudios Agroecológicos y Botánicos, Valparaíso 2340000, Chile; 4 Millennium Nucleus of Patagonian Limit of Life (LiLi), Valdivia 5090000, Chile

**Keywords:** biological invasions, dispersal disruption, frugivory, invasive species, plant–animal mutualism, seed predation, zoochory

## Abstract

Exotic species are one of the main threats to biodiversity, leading to alterations in the structure and functioning of natural ecosystems. However, they can sometimes also provide ecological services, such as seed dispersal. Therefore, we assessed the ecological impacts of exotic species on native dispersal systems and the mechanisms underlying the disruption of mutualistic plant–disperser interactions. Exotic species negatively affect dispersal mutualisms by (i) altering dispersal behavior and visitation rates of native dispersers, (ii) predating native dispersers, (iii) transmitting forest pathogens, and (iv) predating seeds. Conversely, positive impacts include the dispersal of native plants, forest regeneration, and native habitat restoration via (i) increasing the visitation rates of frugivorous birds, (ii) facilitating the colonization and establishment of native forest trees, (iii) enhancing forest species seedling survival, and (iv) facilitating seed rain and seedling recruitment of early and late successional native plants. The reviewed studies provide similar results in some cases and opposite results in others, even within the same taxa. In almost all cases, exotic species cause negative impacts, although sometimes they are necessary to ensure native species’ persistence. Therefore, exotic species management requires a comprehensive understanding of their ecological roles, since the resulting effects rely on the complexity of native–exotic species interactions.

## 1. Introduction

Biological invasions represent one of the main threats to biodiversity, leading to alterations in the structure and functioning of natural ecosystems [1]. Globalization has weakened biogeographic barriers to dispersal, facilitating species introduction into a wide range of new habitats [2]. These introductions have directly and indirectly altered mutualistic interactions, such as pollination and seed dispersal [3,4]. Mutualistic interactions between plants and animals are key for ecosystem functioning, since most plant species depend on biotic vectors for reproduction and recruitment [5], with 90% of plants dispersed by animals in tropical regions and 60% in temperate regions [6]. In addition, seed dispersal maintains gene flow among populations and facilitates recruitment and seedling establishment [7]. Therefore, alterations of dispersal networks by exotic species can change species community composition and collapse the regeneration of several plant populations, leading to local extinctions [8].

Exotic animal species are known to affect mutualistic interactions, such as pollination and seed dispersal, the competitive exclusion of native species being one of the most important mechanisms of interaction disruption [3]. Such disruption is particularly important since seed dispersers play a major ecological role in maintaining community composition and diversity [9]. Consequently, disrupting plant–disperser mutualisms can lead to coextinctions, altering plant community structure and promoting novel interactions between exotic and native species [4,10]. Nevertheless, the impacts of exotic species on mutualistic seed dispersal interactions are context-dependent, leading to contrasting results across taxa and habitats [11].

Several exotic animal species can outcompete native dispersers or affect their dispersal behavior [12,13]. They may also facilitate the introduction of exotic plants by dispersing them and enhancing seedling recruitment success [14,15,16]. Additionally, these species behave as fruit consumers and seed predators in their introduced ranges, affecting plant recruitment and seed bank availability [17], and also disrupting the mutualistic plant–disperser interactions by limiting food resource availability [18,19]. However, in addition to the negative effects of exotic species, in many cases they provide desirable ecological functions, such as the seed dispersal of native species when the original dispersers have become extinct or are rare [20]. Moreover, exotic species may facilitate the attraction of frugivorous species that favor dispersal services [21,22], and even enhance the regeneration of degraded habitats [23]. Therefore, there is certain ambiguity when evaluating the impacts of exotic species, although these are not always negative.

This systematic review aims to elucidate the ecological impacts of exotic species on native dispersal systems by examining the available published peer-reviewed literature. We aimed to provide a qualitative assessment that examined the effects of exotic animals on the dynamics of dispersal and predation of native fruits and seeds. We also examined the mechanisms underlying the disruption of mutualistic plant–disperser interactions mediated by exotic species. In addition, we aimed to highlight conflicting results derived from different study models and geographic regions to find common issues that allow a better understanding of the ecological impacts of exotic species on mutualistic interactions.

## 2. Results

### 2.1. General Overview

**Figure 1:**
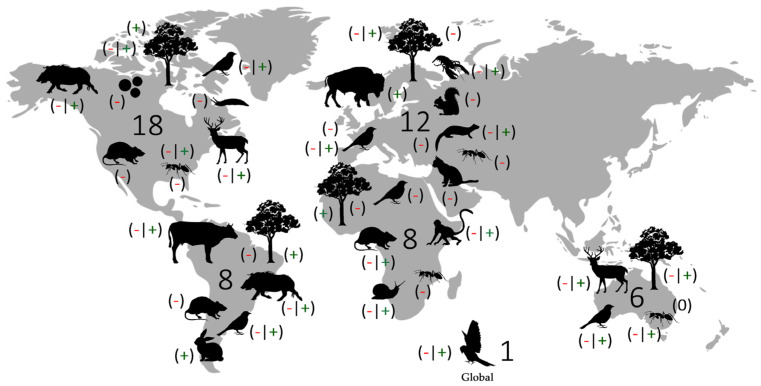
General overview of exotic taxa distribution and their ecological impacts on native seed dispersal systems, including the number of studies reviewed for each continent. Symbols indicate the effect exerted by exotic species, classified as negative (−), positive (+), mixed (−|+), and neutral (0).

Most studies were conducted within the last two decades (from 2001 to 2022) in North America and Europe (n = 18 and 12, respectively), followed by South America and Africa (n = 8 in both cases), Oceania (n = 6), and on a global scale (n = 1) (Figure 1). About 70% of the reviewed articles were focused on the ecological impacts of exotic species as disruptors of plant–disperser mutualisms and as dispersers of native plant seeds (n = 19 and 18 studies, respectively). Exotic animals include a wide variety of mammal taxa such as lagomorphs, rodents, bovids, and feral cats, which interact with plants and thus exert direct and indirect impacts on native seed dispersal systems. Additionally, some studies evaluated the impacts of exotic birds, ants, slugs, arthropods, exotic plants, and tree pathogens, with a few cases reporting combined effects of different taxa. Dispersal systems include mainly endozoochory and myrmecochory, while epizoochory and larder and scatter hoarding are less common, with only one study targeting diplochory for an exotic mammal species. Most of the studies were conducted in the field, assessing the impacts of frugivorous species and ants with a higher frequency (Appendix A).

### 2.2. Dispersal of Native Plant Species

From the examined studies, 18 reported the role of exotic species as seed dispersers of native plants in their introduced ranges. These studies were mainly conducted in South America (n = 5), followed by North America (n = 4,), Europe and Oceania (n = 3 in both cases), Africa (n = 2), and on a global scale (n = 1, including 48 countries from 5 continents), and included mainly mammals (n = 9), followed by birds (n = 6), and ants, mollusks, and freshwater arthropods (n = 1 in all 3 cases) (Table 1). Within the exotic mammals considered legitimate dispersers of native seed plants are the European rabbit, deer, wild boar, birds, domestic cattle, pine martens, and the European bison, which are all terrestrial endozoochoric species. However, most species were reported to have negative ecological effects, such as the dispersal of exotic species, plant invasion facilitation, native seed destruction, native dispersers’ population decline, and herbivory.

Therefore, almost all these cases were considered to have both positive and negative effects simultaneously (−/+), with one as positive (+) since the potential negative ecological impacts of the species (e.g., dispersal of exotic species) were not evaluated.

Since exotic species can disperse the seeds of both native and exotic plants, it is necessary to ponder their ecological effects. For example, the European rabbit is a legitimate seed disperser of a native forest species, and it also favors seedling recruitment [35]; however, this exotic mammal is widely known to disperse seeds of invasive plant species (e.g., [37,38,39]), in addition to grazing on tree seedlings of native species [40,41]. Similarly, the long-tailed macaque disperses but also destroys native seeds, and facilitates plant invasions [25], while exotic birds can disperse both native and exotic plants species via epi- and endozoochory [27,36]. Other exotic birds can surrogate dispersal services in depauperated frugivory communities, but their reduced efficiency can lead to low germination rates and increased pre-dispersal predation in undispersed seeds of native plants [31].

Furthermore, reduced populations of exotic ungulates can have neutral or positive effects on native tree sapling abundance by dispersing seeds, but large populations can result in overgrazing, hampering long-term habitat persistence [32]. In other studies, giant snails are thought to restore recruitment by feeding on fruit flesh of native plants, but snails are widely acknowledged as important herbivores [24]. On the other hand, exotic ants increased seed dispersal ranges of native species and presumably reduce seed predation; however, they may also be involved in the potential decline in native ant diversity [28]. Since these arthropods play an important role in trophic dynamics, ant diversity loss can lead to negative ecological consequences at multiple levels. Thus, even though exotic species can provide dispersal services in defaunated ecosystems or when dispersal by native species has been reduced, their positive and negative interactions with local species must be considered to develop adequate management of invasive plants and animals.

### 2.3. Forest Regeneration and Restoration of Degraded Habitats

Invasive species are one of the main drivers of biodiversity change [42], leading to alterations in the structure and functioning of natural ecosystems [1]. It is widely recognized that exotic animal species could alter seed dispersal services and limit seed bank availability and seedling recruitment through different mechanisms. However, eight of the reviewed studies (Table 2) showed that animal and plant species can contribute to forest regeneration and restoration of degraded habitats. This ecological role was considered as a positive impact in all the cases (+), except for two cases that also involved the dispersal of exotic plant species and outcompeting native plants, which were considered as (−/+). These studies were focused on the effects of exotic frugivore species and the role of exotic plants as seed dispersers attractors for native plants from Africa (n = 2), North America (n = 2), Europe (n = 1), South America (n = 2), and Oceania (n = 1).

The potentials of exotic species for forest regeneration and restoration of degraded habitats are explained by different mechanisms (Table 2). For example, exotic fruit trees have been shown to facilitate seed rain and seedling recruitment of early and late successional native plant species, showing great potential for forest restoration management [23,46]. Furthermore, fruit trees in abandoned farmlands serve as seed disperser attractants, and facilitate seedling recruitment of forest native species [21]. Similarly, exotic plants can increase the visitation rates of frugivorous birds and enhance seed dispersal and seedling establishment processes [44,45]. On the other hand, some exotic frugivores disperse native species, contributing to the restoration of degraded habitats or enhancing the seedling survival of forest species [22,35,43].

Even though a few exotic species have been observed to be capable promoting forest regeneration, it is necessary to have a comprehensive understanding of ecosystem functioning to consider the potential indirect effects of exotic plants and animals. For example, the disproportionate dispersal of a few native plants by exotic species may lead to biotic homogenization. Most studies examined here are short- or medium-term, and do not evaluate the role of exotic dispersed plants associated with native ones. Thus, more empirical evidence is needed to properly understand these processes.

### 2.4. Alteration and Disruption of Seed Dispersal Mutualisms

**Figure 2:**
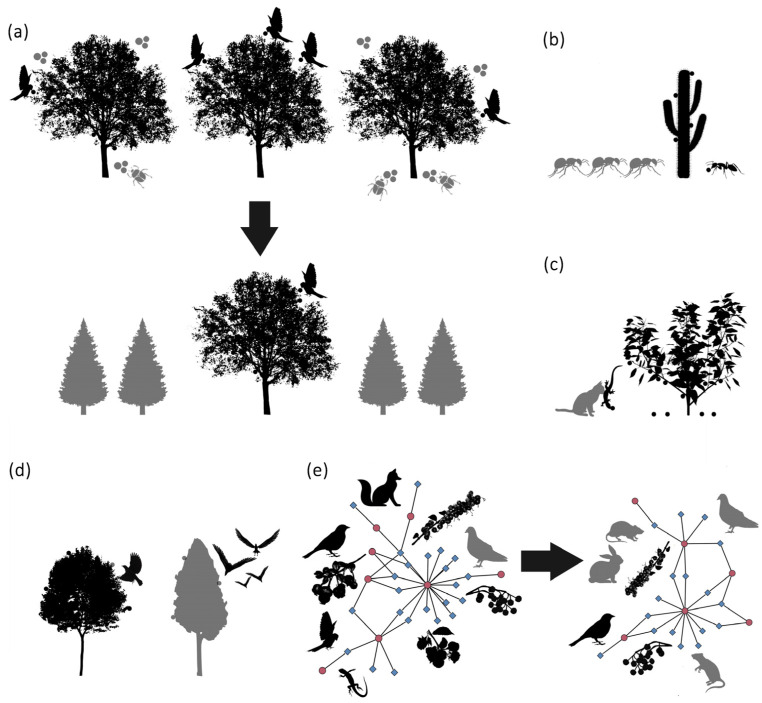
Mechanisms explaining the disruption of plant–disperser mutualism, based on reviewed studies: (**a**) forest pathogens transmitted by exotic species cause a decrease in the population of native trees, with the consequent loss of plant–frugivore interaction and facilitating the invasion of exotic plants; (**b**) exotic species affect dispersal behaviors and visitation rates of native dispersers; (**c**) feral cats predate native frugivorous lizards, disrupting seed dispersal services; (**d**) exotic plants compete with natives for frugivore species; and (**e**) exotic plants negatively influence disperser diversity and alter dispersal networks. Exotic species are represented in gray, and natives in black.

From the reviewed studies, 19 focused on the impacts of exotic species by altering or disrupting plant–disperser mutualisms, mainly in Europe and North American systems (n = 7 in both cases), and also in Africa (n = 4) and Oceania (n = 1). In all the cases, exotic species caused diverse but always negative ecological impacts (−) through different mechanisms. Birds, ants, and feral cats were identified as the main seed dispersal disruptors by affecting primary and secondary dispersal systems. Moreover, exotic plants affect dispersal mutualisms by competing for dispersal services with native plants or reducing disperser diversity. The loss of mutualistic interactions can affect a wide array of ecosystem processes [47], the persistence of plant species, and the structure of plant communities [4]. Thus, the disruption of seed dispersal mutualisms in native communities due to the introduction of exotic species can lead to local extinctions (as well as functional extinctions) and losses of key ecological interactions for ecosystem functioning. The mechanisms explaining the alteration and disruption of plant–disperser mutualisms, based on the reviewed studies, are (a) forest pathogens transmitted by exotic species cause a decrease in the population of native trees, with the consequent loss of plant–frugivore interaction and facilitating the invasion of exotic plants; (b) exotic species outcompete or affect dispersal behaviors and visitation rates of native dispersers; (c) exotic species predate native frugivores, disrupting seed dispersal services; (d) exotic plants compete with natives for frugivore species; and (e) exotic plants negatively influence disperser diversity and alter dispersal networks (Figure 2).

Seed dispersal disruption caused by exotic species in the case studies examined depended on different mechanisms that affect mutualistic interaction between native plants and their dispersers. Sometimes, the disruption of plant–disperser mutualisms relies on more than one species, as in the case of the exotic forest pathogens causing laurel wilt disease in many native Lauraceae species from the USA. These species are responsible for the decline in native plant populations and the consequent disruption of the frugivorous interaction between birds and plants, facilitating the invasion of other exotic plants [48]. Other mechanisms include displacing or affecting dispersal behaviors and visitation rates of native dispersers [4,12,13,18,49,50,51,52,53,54,55,56], modifying patterns of habitat selection by seed dispersers [19,57], predation of native dispersers [58], competition for dispersal services by exotic plants [59,60], and reduction in seed dispersal network complexity [61].

### 2.5. Seed Predation

Although several exotic species are legitimate dispersers of native seed plants in their introduced new ranges see [20,23,29,30,35], many others are considered predators as they exert significant seed damage through consumption or manipulation. In nine of the ten examined studies, seed predation was considered a negative impact (−), since it caused detrimental effects for native plants and, in some cases, also implied the disruption of dispersal processes and seedling recruitment failure (Table 3). In one case, exotic ants were reported to remove seeds of native species, but since seed fate was not evaluated, we categorized it as (0). On the other hand, in an exceptional case, exotic rodents were reported to have a double role by predating and secondary dispersing a native plant species (−|+) (Appendix A). These studies reveal that ants and slugs as well as free-ranging domestic and wild exotic mammals (e.g., goats, horses, cows, pigs, rodents, and rabbits), eat seeds without effectively dispersing them. These studies focused on seed predation by plant species with different dispersal syndromes from North America (n = 5), South America (n = 2), Europe (n = 1), and Oceania and Africa (n = 1 in both cases).

In myrmecochorous systems, seed damage includes not only destruction by consumption, but also by elaiosome detachment, as in the case of the red imported fire ant (*Solenopsis invicta*). Myrmecochorous plants produce seeds with appendages (called elaiosomes) to attract ants that disperse seeds to the subsoil [70]; thus, elaiosome robbing implies a potential disruption of the seed dispersal mutualisms, and can increase seed predation by exposing seeds to other predators on the surface [71]. Similarly, exotic rodents are known to be important seed predators, especially in defaunated ecosystems where native dispersers have become extinct [72]. While native rodents often act as surrogate seed dispersers of extinct species by scatter hoarding mechanisms [73], exotics rodents are usually avid seed predators in invaded habitats, limiting the availability of seed banks and reducing seedling recruitment, as in the case of the black rat *Rattus rattus* [Rattus rattus, 17,24,69]. Additionally, exotic rodents can preclude forest restoration by predating seeds of native plant species [63].

Although exotic rodents can disperse seeds of exotic plant species, they can play a double role by behaving simultaneously as seed dispersers and predators of native plant species [62]. Lastly, exotic mammals can decrease native seed availability and the number of seedlings [68], and compete with native dispersers [69]. Thus, they can trigger a cascade of ecological effects, resulting in the decline in native plant species, the alteration of the structure and composition of the invaded habitat, and the loss of mutualistic interactions.

## 3. Materials and Methods

**Figure 3:**
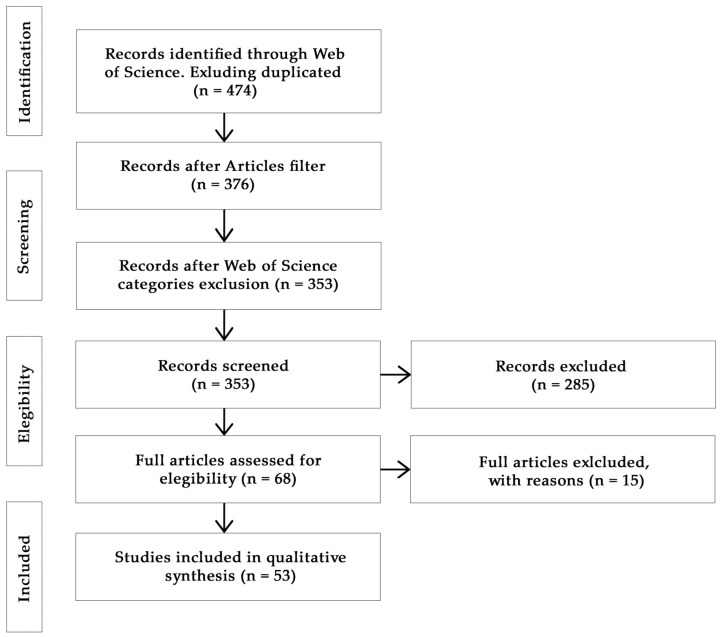
PRISMA flowchart summarizing the sequence of information gathering and selection for the systematic review.

We searched the available literature through the Web of Science database (from January 1975 to October 2022), using search terms related to seed ‘dispersal’, ‘dispersion’, or ‘dispersing’ (seed dispers*), exotic species (exotic OR alien OR invasive OR non-native OR naturalized OR introduced OR non-indigenous), and their impacts on seed dispersal systems (impact* OR effect* OR disrupt*). This initial search returned 474 articles, which were refined by categories; those articles on geography, anthropology, sociology, and other similar fields were considered irrelevant and excluded. After performing this initial filter, we obtained 376 articles, to which we applied a new filter by selecting only research articles (i.e., removing reviews, book chapters, and early works), resulting in 353 articles. Then, we examined these articles by looking for abstracts that met the following criteria: (i) disruption of native seed dispersal systems by exotic species, (ii) seed damage or seed predation of native species by exotic animals, (iii) seed dispersal of native species by exotic animals, and (iv) ecological impacts of exotic species on habitat restoration. Based on the abstract selection, 68 studies were considered for full-article review. Those articles focused on the dispersal of exotic plant species, but excluding natives, as well as those that did not evaluate the ecological impacts of exotic species on native seed dispersal systems, were not considered. Article selection and review process followed the PRISMA Statement guidelines [74]. Finally, 53 studies were included for systematic review (Figure 3).

The studies selected were classified according to the ecological impacts that exotic species exert on the native seed dispersal systems either as negative (−), if the species alter or disrupt native plant–disperser mutualisms, predate or damage native seeds, or facilitate seedling recruitment and establishment of exotic plants; positive (+), if the species are legitimate seeds dispersers of native plants, enhance the visitation rates of native dispersers, or contribute to forest regeneration and restoration of degraded habitats; mixed (−|+), when positive and negative ecological impacts are simultaneously reported; or neutral (0), if there is a not clear ecological impact of exotic species in positive or negative directions. This categorization was based on the reported results from the reviewed studies, from which we also extracted data regarding location, exotic taxa involved, and underlying mechanisms.

## 4. Conclusions and Perspectives

The ecological impacts of exotic species on the seed dispersal systems of native species widely varied, with significant negative and positive effects on the maintenance of plant–disperser mutualisms. The reviewed articles offer different methodological approaches based on different taxa, as well as across dispersal syndromes and among geographic ranges, showing rather similar results in some cases and opposite outcomes in others, even when the same taxa were evaluated. The possible ecological outcomes derived from native–exotic species interactions must be comprehensively evaluated, especially for exotic species management, due to the complexity of facilitation relationships. Exotic species can play critical roles in ecosystem functioning by performing dispersal services; thus, removing them can lead to regeneration collapses. Nevertheless, they can also disrupt mutualist interactions, and extirpations can be necessary to avoid local extinctions. Additionally, sometimes species dynamics can be more complex, and exotic species must be controlled instead of removed, i.e., reducing or maintaining population sizes to diminish negative impacts on native communities. Hence, contrasting impacts must be pondered from case to case, considering both positive and potential negative effects. The classic paradigm that only exotic species cause negative ecological impacts must be confronted, as this generalization precludes the fact that different species can exert different effects on plant communities, which is sometimes necessary to ensure native species’ persistence.

## Figures and Tables

**Table 1:** Summary of the exotic taxa involved in native seeds dispersal and their additional ecological impacts.

Location	Native Plants Dispersed	Exotic Taxa	Associated Negative Ecological Impacts	Reviewed Studies
Africa (Mauritius)	*Labourdonnaisia calophylloides*, *Mimusops balata* *	*Lissachatina immaculata*	Potential herbivory	[24]
Africa (Mauritius)	Several species	*Macaca fascicularis*	Native seed destruction, potential plant invasion facilitation	[25]
Europe (Poland)	*Quercus robur*	*Garrulus glandarius*	Potential positive impacts for exotic plant populations	[15]
Europe (Balearic Islands, Spain)	Several species	*Martes*	Potential seed dispersal mutualism disruption	[26]
Europe (Spain)	Several species	*Procambarus clarkii*	Dispersal of invasive species	[16]
North America (Hawai’i, USA)	Several species	*Leiothrix lutea*, *Pycnonotus jocosus*, *P. cafer*, *Zosterops japonicus*	Dispersal of invasive species	[27]
North America (USA)	Several species	*Myrmica rubra*	Potential decline in the diversity of native ants	[28]
North America (USA)	Several species	*Odocoileus virginianus*	Reduced reproductive output, dispersal of exotic plant species	[14]
North America (Mariana Island, USA)	Several species	*Rusa marianna*, *Sus scrofa*	Decrease in seedling abundance	[29]
Oceania (Australia)	Several species	*Axis porcinus*	Dispersal of exotic plant species	[30]
Oceania (New Zealand)	Several species	Birds	Potential alteration of the structure of plant–seed disperser assemblages	[11]
Oceania (New Zealand)	*Pittosporum crassifolium*	Birds	Low germination and high pre-dispersal predation in undispersed seeds	[31]
South America (Argentina)	*Prosopis flexuosa*	*Bos primigenius taurus*, *Equus africanus asinus*, *E. ferus caballus*, *E. mulus*	Potential overgrazing at high population densities	[32]
South America (Argentina)	Several species	*Lophura nycthemera*	Dispersal of invasive plants species	[33]
South America (Brazil)	Several species	*Sus scrofa*	Dispersal of exotic plant species	[20]
South America (Brazil)	Several species	*Sus scrofa*	Dispersal of exotic plant species	[34]
South America (Chile)	*Lithrea caustica*	*Oryctolagus cuniculus*	Not reported	[35]
Global	Several species	Parrots	Potential plant invasion	[36]

* Plants are not formally dispersed, but exotic species positively affect their recruitment by feeding on fruit flesh.

**Table 2:** Exotic taxa involved in forest regeneration and restoration of degraded habitats processes.

Location	Exotic Taxa	Mechanisms	Reviewed Studies
Africa (Kenya)	*Psidium guajava*	(i) Fruit trees facilitate seed rain and seedling recruitment of early and late successional native plants	[23]
Africa (Uganda)	*Persea americana*, *Mangifera indica*, *Eucalyptus* sp.	(ii) Fruit trees facilitate the colonization of native forest on abandoned farmlands through the dispersal and establishment of native tree species	[21]
Europe (Poland)	*Bison bonasus* *	(iii) Frugivores disperse native species contributing to the restoration of degraded habitats or enhanced seedling survival of forest species	[43]
North America (Santa Cruz Island, USA)	*Foeniculum vulgare*	(iv) Exotic plants increase visitation rates by frugivorous birds, consequently increasing seed dispersal and establishment	[44]
North America (USA)	*Lonicera* spp.	(iv) Exotic plants increase visitation rates by frugivorous birds, consequently increasing seed dispersal and establishment	[45]
Oceania (Australia)	*Cinnamomum camphora*	(v) Frugivores disperse native species contributing to the restoration of degraded habitats or enhanced seedling survival of forest species	[22]
South America (Chile)	*Oryctolagus cuniculus*	(iii) Frugivores disperse native species contributing to the restoration of degraded habitats or enhanced seedling survivor of forest species	[35]
South America (Brazil)	*Artocarpus heterophyllus*	(i) Fruit trees facilitate seed rain and seedling recruitment of early and late successional native plants	[46]

* *Bison bonasus* became extinct in the wild and has been reintroduced in some areas of Central and Eastern Europe.

**Table 3:** Summary of the exotic taxa involved in native seeds predation and their additional ecological impacts.

Location	Native Plants Dispersed	Exotic Taxa	Associated Negative Ecological Impacts	Reviewed Studies
Africa (Mauritius)	Zoochory	*Rattus rattus*	Not evaluated	[24]
Europe (Canary Islands, Spain)	Several	*Atlantoxerus getulus*, *Oryctolagus cuniculus*	Frugivore–plant mutualism disruption	[62]
North America (Hawai’i, USA)	Unknown	*Rattus rattus*	Not evaluated	[63]
North America (USA)	Myrmecochory	*Solenopsis invicta*	Not evaluated	[64]
North America (USA)	Several	*Rattus rattus*	Not evaluated	[65]
North America (Hawai’i, USA)	Unknown	*Rattus rattus*	Seedling recruitment failure	[17]
North America (Canada)	Myrmecochory	*Myrmica rubra*, *Arion subfuscus*	Ant–plant mutualism disruption	[66]
Oceania (Australia)	Myrmecochory	Ants	Not evaluated	[67]
South America (Chile and Argentina)	Several	Free-ranging domestic and wild exotic mammals	Reduced seedling survival, mutualism disruption	[68]
South America (Chile)	Scatter hoarding	*Rattus rattus*	Competition with native rodent dispersers	[69]

## Data Availability

Not applicable.

## Appendix A

**Table A1:** List of reviewed articles, including exotic taxa involved in each study and their ecological impacts and mechanisms.

Source	Tittle	Location	Exotic Taxa	Mechanisms and Impacts
[24]	Tree recruitment after native frugivore extinction? A field experiment to test the impact of fruit flesh persistence in a tropical oceanic island	Africa (Mauritius)	*Lissachatina immaculata*; *Rattus rattus*	(−|+) Potential herbivory (by *L. immaculata*; seed dispersal (by *L. immaculata* and *R. rattus*)
[31]	Effects of changes in bird community composition and species abundance on plant reproduction, through pollination and seed dispersal	Oceania (New Zealand)	*Pittosporum crassifolium*	(−|+) Low germination and high pre-dispersal predation in undispersed seeds; seed dispersal
[23]	Exotic guavas are foci of forest regeneration in Kenyan farmland	Africa (Kenya)	*Psidium guajava*	(+) Forest regeneration, contribution to native seed dispersal and seedling recruitment
[67]	Biodiversity impacts of an invasive grass: ant community responses to *Cenchrus ciliaris* in arid Australia	Oceania (Australia)	*Cenchrus ciliaris*, ants	(0) Seed removal by ants with unknown effects
[32]	Environmental and biological factors affecting the abundance of *Prosopis flexuosa* saplings in the central-west Monte of Argentina	South America (Argentina)	*Bos primigenius taurus, Equus africanus asinus, E. ferus caballus*, *E. mulus*	(−|+) Potential overgrazing at high population densities; seed dispersal
[35]	Ecological efficiency and legitimacy in seed dispersal of an endemic shrub (*Lithrea caustica*) by the European rabbit (*Oryctolagus cuniculus*) in central Chile	South America (Chile)	*Oryctolagus cuniculus*	(+) Seed dispersal, seedling survival enhanced
[26]	Contrasting patterns of seed dispersal between alien mammals and native lizards in a declining plant species	Europe (Balearic Islands, Spain)	*Martes martes*, *Cneorum tricoccon*	(−|+) Potential seed dispersal mutualism disruption; seed dispersal
[12]	Consequences of a biological invasion reveal the importance of mutualism for plant communities	Africa (South Africa)	Ants	(−) Seed dispersal disruption by eliminating native-ant dispersers for large-seeded species
[17]	Could poor seed dispersal contribute to predation by introduced rodents in a Hawaiian dry forest?	North America (USA)	*Rattus rattus*	(−) Seed predation and seedling recruitment failure
[48]	Bird–plant interactions and vulnerability to biological invasions	North America (USA)	*Xyleborus glabratus*, *Raffalea lauricola*	(−) Disruption of plant–frugivore mutualism by declining plant populations
[69]	Multiple anthropogenic pressures lead to seed dispersal collapse of the southernmost palm *Jubaea chilensis*	South America (Chile)	*Rattus rattus*	(−) Competition with native rodent dispersers
[59]	Seed dispersal by frugivores from forest remnants promotes the regeneration of adjacent invaded forests in an oceanic island	Africa (Seychelles)	*Cinnamomum verum*, *Clidemia hirta*	(-|+) Competition for dispersal services; seed dispersal
[60]	Seasonal variation in impact of non-native species on tropical seed dispersal networks	Africa (Seychelles)	Several taxa	(−) Competition for dispersal services
[30]	Facilitative interactions between an exotic mammal and native and exotic plants: hog deer (*Axis porcinus*) as seed dispersers in south-eastern Australia	Oceania (Australia)	*Axis porcinus*	(−|+) Seed dispersal of exotic and native plant species
[18]	Invasive ants take and squander native seeds: implications for native plant communities	Europe (Iberian Peninsula)	*Linepithema humile*	(−) Seed dispersal service reduction by mutualism disruption
[28]	Impacts of the Invasive European Red Ant (*Myrmica rubra* (L.): Hymenoptera; Formicidae) on a Myrmecochorous System in the Northeastern United States	North America (USA)	*Myrmica rubra*	(−|+) Potential decline in native ant diversity; increased seed dispersal range, potential seed predation reduction
[11]	Exotic birds increase generalization and compensate for native bird decline in plant–frugivore assemblages	Oceania (New Zealand)	Birds	(−|+) Potential alteration of the structure of plant–seed disperser assemblages; maintained frugivory when native birds became rarer
[29]	Contrasting ecological roles of non-native ungulates in a novel ecosystem	North America (USA)	*Rusa marianna*, *Sus scrofa*	(−|+) Decrease in native seedling abundance; seed dispersal
[45]	Fruit quantity of invasive shrubs predicts the abundance of common native avian frugivores in central Pennsylvania	North America (USA)	*Lonicera* spp.	(+) Enhanced dispersal and establishment of native species
[46]	Jackfruit trees as seed attractors and nurses of early recruitment of native plant species in a secondary forest in Brazil	South America (Brazil)	*Artocarpus heterophyllus*	(+) Facilitation of seed rain and seedling recruitment of early and late successional native plant species
[65]	Husking stations provide insight into diet of nonnative rodents on O‘ahu, Hawai‘i	North America (USA)	*Rattus rattus*	(−) Seed predation
[51]	Integration of exotic seeds into an Azorean seed dispersal network	Europe (Portugal)	Birds	(−) Reduced the number of native seeds dispersed by diverting consumers of native plants
[36]	Epizoochory in parrots as an overlooked yet widespread plant–animal mutualism	Global	*Parrots*	(−|+) Potential plant invasion; seed dispersal
[21]	Non-native fruit trees facilitate colonization of native forest on abandoned farmland	Africa (Uganda)	*Persea americana*, *Mangifera indica*, *Eucalyptus*	(+) Facilitation of dispersal and establishment of native tree species
[43]	The European bison as seed dispersers: the effect of the species composition of a disturbed pine forest community	Europe (Poland)	*Bison bonasus*	(+) Dispersal of native species leading to restoration of degraded habitats
[62]	Effects of the invasive Barbary ground squirrel (*Atlantoxerus getulus*) on seed dispersal systems of insular xeric environments	Europe (Spain)	*Atlantoxerus getulu*	(−) Seed damage, mutualism disruption
[16]	Crayfish invasion facilitates dispersal of plants and invertebrates by gulls	Europe (Spain)	*Procambarus clarkia*	(−|+) Seed dispersal of invasive and native species
[33]	Non-redundancy in seed dispersal and germination by native and introduced frugivorous birds: implications of invasive bird impact on native plant communities	South America(Argentina)	*Lophura nycthemera*	(−|+) Seed dispersal of invasive and native species
[66]	An invasive slug exploits an ant–seed dispersal mutualism	North America (Canada)	*Myrmica rubra*, *Arion subfuscus*	(−) Seed damage, ant–plant mutualism disruption
[19]	Invasive ants disperse seeds farther than native ants, affecting the spatial pattern of seedling recruitment and survival	North America (Canada)	Ants	(−) Alteration of the spatial pattern of seedling recruitment and plant survival
[53]	Seed Removal Increased by Scramble Competition with an Invasive Species	North America (USA)	Rodents	(−) Competition between native and exotic species for seed removal, affecting the magnitude and differential spatial patterns of seed removal
[52]	Frugivorous birds visit fruits of emerging alien shrub species more frequently than those of native shrub species in the South African Mediterranean climate region	Africa (South Africa)	Birds	(−) Reduced visitations of fruits by frugivorous birds
[22]	Do frugivorous birds assist rainforest succession in weed dominated old field regrowth of subtropical Australia?	Oceania (Australia)	*Cinnamomum camphora*	(−|+) Recruitment of exotic species; forest regeneration, contribution to native seed dispersal and seedling recruitment
[63]	Removal of non-native trees fosters but alone is insufficient for forest regeneration in Hawai’i	North America (USA)	*Rattus rattus*	(−) Seed predation
[49]	Forest edges and fire ants alter the seed shadow of an ant-dispersed plant	North America (USA)	*Solenopsis invicta*	(−) Mutualist disruption, reduced seed shadow, limited dispersal between types of habitats
[15]	Predation and dispersal of acorns by European Jay (*Garrulus glandarius*) differs between a native (Pedunculate Oak *Quercus robur*) and an introduced oak species (Northern Red Oak *Quercus rubra*) in Europe	Europe (Poland)	*Garrulus glandarius*, *Quercus rubra*	(−|+) Potential positive impacts for exotic plant populations; seed dispersal
[58]	The unnoticed effect of a top predator on complex mutualistic ecological interactions	Europe (Spain)	Feral cats	(−) Disruption of seed dispersal mutualisms, reduction in seed effectiveness
[44]	Recovery of native plant communities after the control of a dominant invasive plant species, *Foeniculum vulgare*: implications for management	North America (USA)	*Foeniculum vulgare*	(−|+) Displacement of native plants; increased visitation by frugivorous birds and likely increased native seed dispersal
[34]	Seed dispersal effectiveness by a large-bodied invasive species in defaunated landscapes	South America (Brazil)	*Sus scrofa*	(−|+) Seed dispersal of exotic species; dispersal of native seeds in defaunated forests
[20]	Reunion overseas: introduced wild boars and cultivated orange trees interact in the Brazilian Atlantic Forest	South America (Brazil)	*Sus scrofa*	(−|+) Seed dispersal of exotic species; dispersal of native seeds in defaunated forests
[13]	Interactions between seed-dispersing ant species affect plant community composition in field mesocosms	North America (Canada)	*Myrmica rubra*	(−) Alteration of foraging and dispersal behaviors of native dispersers, affecting dispersal and seedling recruitment
[25]	Foraging strategies of invasive *Macaca fascicularis* may promote plant invasion in Mauritius	Africa (Mauritius)	*Macaca fascicularis*	(−|+) Native seed destruction, potential plant invasion facilitation; seed dispersal
[4]	Disruption of ant-seed dispersal mutualisms by the invasive Asian needle ant (*Pachycondyla chinensis*)	North America (USA)	*Pachycondyla chinensis*	(−) Seed dispersal disruption by outcompeting ant native dispersers
[57]	Declining relict plants: climate effect or seed dispersal disruption? A landscape-scale approach	Europe (Balearic Islands, Spain)	*Marten marten*	(−) Seed dispersal disruption, modifying the patterns of habitat selection by plants
[50]	New mutualism for old: indirect disruption and direct facilitation of seed dispersal following Argentine ant invasion	Oceania (Australia)	*Linepithema humile*	(−) Disruption of seed-dispersal mutualisms by displacing natives species, affecting dispersal rates
[61]	Bottom-up cascading effects of quarry revegetation deplete bird-mediated seed dispersal services	Europe (Portugal)	*Pinus halepensis*	(−) Reduction in seed dispersal network complexity
[68]	Large-scale impacts of multiple co-occurring invaders on monkey puzzle forest regeneration, native seed predators and their ecological interactions	South America (Chile and Argentina)	Mammals	(−) Reduced seed availability and seedling survival, disruption of dispersal processes
[54]	Global change drivers synergize with the negative impacts of non-native invasive ants on native seed-dispersing ants	North America (USA)	Ants	(−) Displacement of native dispersers
[55]	Seed removal decrease by invasive Argentine ants in a high nature value farmland	Europe (Portugal)	*Linepithema humile*	(−) Displacement of native dispersers
[27]	Space use patterns and the extent of complementarity across scales in introduced seed dispersers	North America (USA)	*Zosterops japonicus, Leiothrix lutea*, *Pycnonotus jocosus*, *P. cafer*	(−|+) Seed dispersal of invasive species; seed dispersal
[14]	Endozoochory by white-tailed deer (*Odocoileus virginianus*) across a suburban/woodland interface	North America (USA)	*Odocoileus virginianus*	(−|+) Reduced reproductive output of native plants, dispersal of exotic species; dispersal of native plants
[56]	Avian dispersal of an invasive oak is modulated by acorn traits and the presence of a native oak	Europe (Poland)	*Quercus rubra*	(−) Alteration of visitation rates of native dispersers
[64]	Ant–seed mutualisms: can red imported fire ants sour the relationship?	North America (USA)	*Solenopsis invicta*	(−) Seed damage
